# Twiddler’s Syndrome

**DOI:** 10.5334/jbsr.3629

**Published:** 2024-06-03

**Authors:** Vincent Sneyers, Bjorn Valgaeren, Brecht Van Berkel

**Affiliations:** 1 UZ Leuven, Herestraat 49, 3000 Leuven, Belgium; 2 UZ Leuven, Leuven, Belgium; 3 UZ Leuven, Leuven, Belgium

**Keywords:** Twiddler’s syndrome, x-ray, ultrasound, deep brain stimulation

## Abstract

*Teaching point:* Twiddler’s syndrome is a very rare but potentially disastrous complication after implantation of a neurostimulator or pacemaker, caused by twisting of the generator within the subcutaneous pocket, resulting in dislodgement and/or interruption of the electrodes that should be reported on x-ray.

## Case History

**Figure 1:**
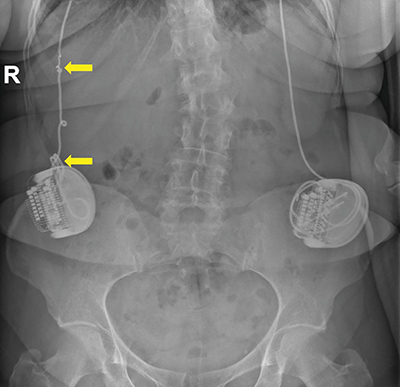
Abdominal x-rays show twisting of the neurostimulator and the electrodes around their axis, on the right side, with an interruption of the right electrode. The left neurostimulator and electrode appear normal.

**Figure 2:**
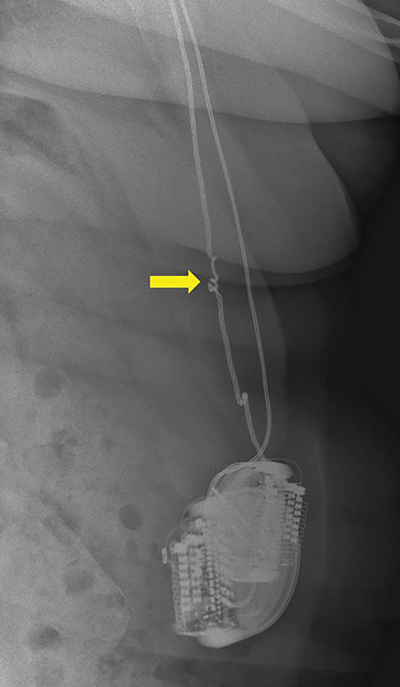
Abdominal x-rays show twisting of the neurostimulator and the electrodes around their axis, on the right side, with an interruption of the right electrode.

**Figure 3:**
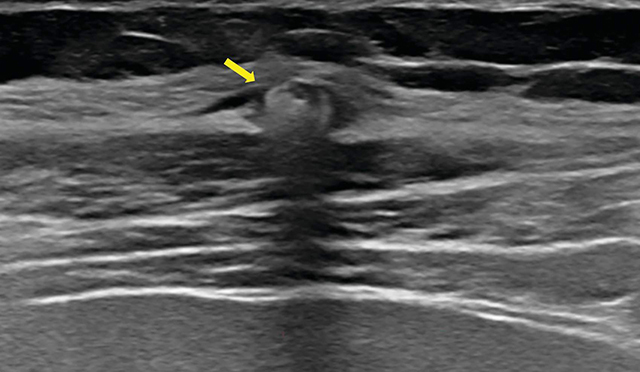
Ultrasound demonstrates some fluid accumulation and focal inflammation of the subcutaneous fat surrounding the affected electrode.

A case of Twiddler’s syndrome is reported in a 64-year-old female with a medical history of obsessive–compulsive disorder (OCD). She underwent bilateral deep brain stimulation (DBS) in the stria terminalis through an abdominally implanted neurostimulator, experiencing positive therapeutic outcomes for over 12 years. More recently, the patient consulted the neurosurgeon with discomfort in the right upper abdominal quadrant and altered mood states. An abdominal x-ray and ultrasound were performed. The radiograph showed twisting of the neurostimulator and the electrodes around their axis and an interruption of the right electrode due to increased tension (Figures 1 and 2). The neurostimulator and electrode course on the left side appeared normal. Ultrasound demonstrated some fluid accumulation and focal inflammation of the subcutaneous fat surrounding the affected electrode (Figure 3), while no abdominal pathology was detected. A surgical intervention with the adjustment of the neurostimulator and electrode on the right side was performed.

## Comments

Twiddler’s syndrome refers to the permanent malfunction of a pacemaker or neurostimulator. This rare complication typically occurs when patients consciously or subconsciously rotate the pulse generator within the subcutaneous pocket, leading to the dislodgement of leads as they wind around the stimulator. Dislocation of the stimulator and fracture of the leads can result from this manipulation [1]. Twiddler’s syndrome typically manifests within the first year after implantation but can also occur later, as in this case. Elderly and obese patients are at an increased risk due to the presence of loose subcutaneous tissue, which allows for easier twisting of the generator in its pocket.

Preventive surgical measures, including the creation of a small surgical pocket and suturing the device to the fascia, contribute to decrease the risk of manipulation of the position of the generator and lead dislodgement. Twiddler’s syndrome is particularly cumbersome in patients with cardiac defibrillators, as false treatment of ventricular arrhythmias and inappropriate shocks may occur due to inadequate sensing and capture.

Besides patient history and clinical examination, x-rays and pacemaker/neurostimulator interrogations serve as standard diagnostic tools for Twiddler’s syndrome. X-ray imaging reveals twisting of the generator along its long or transverse axis, as well as interruption and displacement of the electrodes. Appropriate reporting of Twiddler’s syndrome is essential for the management and prevention of further complications.
